# Biological relevance of CNV calling methods using familial relatedness including monozygotic twins

**DOI:** 10.1186/1471-2105-15-114

**Published:** 2014-04-21

**Authors:** Christina A Castellani, Melkaye G Melka, Andrea E Wishart, M Elizabeth O Locke, Zain Awamleh, Richard L O’Reilly, Shiva M Singh

**Affiliations:** 1 Department of Biology, The University of Western Ontario, London N6A 5B7, ON, Canada; 2 Department of Computer Science, The University of Western Ontario, London N6A 5B7, ON, Canada; 3 Department of Psychiatry, The University of Western Ontario, London N6A 5B7, ON, Canada

**Keywords:** Microarrays, Copy number variation, Genetic relatedness, CNV calling methods, Monozygotic twins

## Abstract

**Background:**

Studies involving the analysis of structural variation including Copy Number Variation (CNV) have recently exploded in the literature. Furthermore, CNVs have been associated with a number of complex diseases and neurodevelopmental disorders. Common methods for CNV detection use SNP, CNV, or CGH arrays, where the signal intensities of consecutive probes are used to define the number of copies associated with a given genomic region. These practices pose a number of challenges that interfere with the ability of available methods to accurately call CNVs. It has, therefore, become necessary to develop experimental protocols to test the reliability of CNV calling methods from microarray data so that researchers can properly discriminate biologically relevant data from noise.

**Results:**

We have developed a workflow for the integration of data from multiple CNV calling algorithms using the same array results. It uses four CNV calling programs: PennCNV (PC), Affymetrix^®^ Genotyping Console™ (AGC), Partek^®^ Genomics Suite™ (PGS) and Golden Helix SVS™ (GH) to analyze CEL files from the Affymetrix^®^ Human SNP 6.0 Array™. To assess the relative suitability of each program, we used individuals of known genetic relationships. We found significant differences in CNV calls obtained by different CNV calling programs.

**Conclusions:**

Although the programs showed variable patterns of CNVs in the same individuals, their distribution in individuals of different degrees of genetic relatedness has allowed us to offer two suggestions. The first involves the use of multiple algorithms for the detection of the largest possible number of CNVs, and the second suggests the use of PennCNV over all other methods when the use of only one software program is desirable.

## Background

Copy number variants (CNVs) are defined as DNA segments (often 50 bp or larger) that are present in variable numbers in a genome [1-3]. Although common in the human genome, some CNVs have no apparent phenotypic effect [1,4,5], while others are implicated in a variety of phenotypic effects including disease phenotypes [6-8]. As such, the search for CNVs associated with disease phenotypes has emerged as a productive approach to identify genetic factors underlying a number of common and complex neurodevelopmental disorders [9-13]. There are two reasons for this productivity. Firstly, CNVs are a major contributor to genomic variation, with approximately 13% of the human genome affected by CNVs [5], and over 100,000 CNVs have been mapped to specific genomic locations and are documented in the Database of Genomic Variants (DGV) [14]. Secondly, advances in technology, including microarrays, permit high-throughput methods to identify CNVs. Such technologies are now relatively common and are economically feasible alternatives to methods like whole genome sequencing. With a number of array platforms and bioinformatic algorithms available, it is necessary to identify optimal analytical pipelines to make inferences regarding specific genomic regions and their copy number identity.

On genome-wide microarrays, such as the Affymetrix^®^ Genome-Wide Human SNP Array 6.0™, sets of probes are designed to determine which allele is present at genomic sites of known single nucleotide polymorphism (SNP probes). The arrays may also include additional probes designed for genomic sites where there is no known variance (known as copy number probes). Normally, CNVs are identified by fluorescent signals generated by SNP probes on the microarray. The fluorescent signals emitted by both SNP probes and copy number probes (if present on the array) are summarized and analyzed for variance in signal intensity using bioinformatic tools, typically in comparison to a set of reference samples. Consecutive markers that exhibit altered signal intensity from the reference are interpreted as copy number variants (CNVs). There are a number of algorithms that have been developed to identify putative CNVs. Unfortunately, not all putative CNVs called by any existing algorithm can be viewed as biologically relevant. The application of multiple software programs that are designed to call CNVs from the same microarray data often yield differing results [7,15-18]. The use of multiple algorithms has been shown to increase the reliability of observations with different degrees of confidence. For example, Kim et al. used three calling algorithms (PennCNV, QuantiSNP, and Birdsuite) on a set of results from Affymetrix^®^ arrays. They found that only 1.5% of total CNV calls could be identified by all three distinct algorithms [16]. Furthermore, their attempt to confirm putative CNV calls using qPCR produced differing results; 38.3% of CNVs called by a single algorithm, 57.6% of CNVs called by two algorithms and 71.4% of CNVs called by three algorithms could be confirmed by qPCR [16].

Although SNP arrays have become popular for ascertaining copy number data in addition to SNP genotypes, there are many issues intrinsic to the use of SNP arrays for the identification of CNVs. Theoretically, it is possible to resolve some of these issues through the use of sensitive analytical methods. In fact, the past ten years have seen a boom in the development of algorithms and technical resolution that have been applied across platforms and programs [19]. Quality control measures such as batch effect correction, normalisation methods, and reference group choice seem like simple considerations when compared to the choices available in both algorithms and CNV identification software, as well as in post-analysis filters like marker density and minimum marker thresholds. The reality of limited biological validity in the use of microarrays to call copy number variable regions is concerning for this area of research that may hold exceptional promise in clinical applications [20]. The need for best practices in the workflow for CNV calling protocols has never been more essential.

This study is aimed at assessing putative CNV calls made using the Affymetrix^®^ Human SNP 6.0 Array™ using four CNV calling programs: PennCNV [21], Affymetrix^®^ Genotyping Console™ [22], Partek^®^ Genomics Suite™ (Partek Inc., St. Louis, MO, USA), and Golden Helix SNP and Variation Suite™ (Golden Helix, Bonzeman, MT, USA). Using individuals of known relatedness, we have identified overlapping copy number variants across the four algorithms. Using the dataset generated, we have assessed the relative sensitivity of each of the four methods from the following comparisons: between unrelated individuals, between parents and offspring, and between monozygotic twins, that are known to share 0%, 50%, and 100% genetic relatedness, respectively. We argue that the most biologically relevant CNVs will be expected to follow this relationship, with the exception of *de novo* events. The results showed that overall, Affymetrix^®^ Genotyping Console™ identified the most differences between unrelated individuals, while Partek^®^ yielded the most similarity between identical twins. On average, PennCNV called CNVs that were comparable to Affymetrix^®^ Genotyping Console™ across unrelated individuals and CNVs that were similar to the Partek^®^ results. Assessments using Golden Helix did not follow the trends expected from the known genetic relatedness of individuals. We argue that a combination of three programs (Affymetrix^®^ Genotyping Console™, Partek, and PennCNV) may be optimal to identify biologically relevant CNV calls due to their ability to resolve copy number variations across different biological relatedness.

## Methods

This study received ethics approval by the University of Western Ontario’s Committee on Research Involving Human Subjects. Written informed consent was obtained from all participants. A total of 16 genomic DNA samples were isolated from whole blood representing the study participants that included six pairs of MZ twins (three female pairs and three male pairs) and two sets of parents for two of the twin pairs (*n* = 16). The six twin pairs ranged in age from 20 to 53 years at the time of sample collection. Genomic DNA was extracted from whole blood using the PerfectPure™ DNA Blood Kit following the manufacturer’s protocol (Invitrogen, Carlsbad, CA). Whole genome microarray analysis was performed using the Affymetrix^®^ Genome-Wide Human SNP Array 6.0™ at the London Regional Genomics Center (London, ON) following the manufacturer’s protocol. Sixteen arrays (one array per sample) were processed and analyzed as a single batch and scanned to produce CEL files. The CEL files were used to generate CNV calls on all 16 individuals using four programs: Affymetrix^®^ Genotyping Console 4.1.1™ (AGC), Partek^®^ Genomics Suite™ (PGS) PennCNV (PC), and Golden Helix^®^ SVS Suite 7.0™ (GH). In Affymetrix^®^ Genotyping Console, we used both the Birdsuite package (version 2) and the Canary algorithm for CNV detection. Birdseye, which is found in Birdsuite, was used for the detection of rare CNVs via a Hidden Markov Model (HMM) and Canary was used to call copy number state in genomic regions with known copy number polymorphism. In Partek^®^, HMM Region Detection using default parameters was selected. In PennCNV, the default HMM algorithm was selected. In Golden Helix^®^ we used the CNAM optimal segmenting algorithm. HMM-based algorithms use prior probabilities of copy number states in conjunction with array-derived normalized fluorescent intensity values to call the most likely copy number state in a given genomic region. The copy number states determined by HMM are discrete and they include 0, 1, 2, 3, and 4. On the other hand, genomic segmentation scans two adjacent regions of the genome to find differences in copy number using two specific t-tests. CNAM optimal segmenting uses genetic marker map information alongside log2 ratios to discover regions in which log2 ratios vary significantly between adjacent segments. We used the univariate method of optimal segmenting which segments each sample in the study separately. Canary, which was used in Affymetrix Genotyping Console, calculates CNP copy number state for over 1,100 regions of known copy number polymorphism (frequency in the population greater than one percent).

The user-defined analytical parameters were kept consistent across the analysis. Specifically, the HapMap 270 6.0 Array reference was used as a reference file and variants were identified as DNA regions, which were called as copy number state of 0, 1, 3, or 4+ covering a minimum of 10 consecutive markers on the array. Only variants greater than 1 kb in size were included in subsequent analysis. The CNV calls made by each of the four software programs were merged with adjacent CNV calls that may represent the same CNV event. The criteria used to merge were 1) CNVs had to be adjacent on the same chromosome (no other CNV call between them); 2) CNVs had to share the same gain/loss status; and 3) adjacent calls were ≤ 20% of the total length, that is, if there were three consecutive genomic segments A, B and C, where A and C are both losses and B is unchanged, we divided the length of the gap B by the length of A + B + C. If this fraction was ≤20%, then we merged A + B + C as a single CNV call*.* If there were multiple consecutive CNVs, each with 20% or less length between one and all of the others, then we extended the formula to the next CNV and merged all of the CNVs into one event. When multiple smaller CNVs were merged into one large CNV event, we identified the event as a merged CNV. We then labeled our newly merged CNVs and any CNVs that remained unmerged as either “CNV-Gain” or “CNV-Loss” within the calls from all four software programs in all individuals.

To compare CNV calls made by different software programs, we used a 50% reciprocal overlap (RO) criterion to compare the calls made within an individual from the four software programs. The use of 50% RO for comparing calls is consistent with other reports [23-25]. Two CNV events were considered to pass the 50% RO criterion if at least half of the length of the first CNV overlapped with the second CNV and vice versa. If the 50% RO criterion was met, the two events were then considered to be the same event (called by different algorithms but identified in the same individual) as long as their call states also matched. We calculated the reciprocal overlap (O) (≥50% criteria) as follows:

Where x and y are both CNVs, L is length in base pairs that the two CNVs (x and y) overlap, end indicates the end base pair position of the given CNV, and start indicates the start base pair position of the given CNV.

$$O\left(A\right)=\frac{L}{x_{\text{end}}-x_{\text{start}}+1}$$

$$O\left(B\right)=\frac{L}{y_{\text{end}}-y_{\text{start}}+1}$$

CNVs met the ≥50% RO criteria if O(A) and O(B) were both ≥50%. CNVs that did not meet this criterion were considered to be different events.

Finally, the same RO definition (≥50%) was used to compare shared and unshared calls in a pairwise comparison between individuals in the following categories of genetic relatedness: between MZ twins in a twin pair, between parent and child, and between unrelated individuals. Following RO comparisons, we calculated the average difference (*d*) within each group (where *d* is the total number of unshared CNVs across the two compared individuals, divided by the total number of CNVs called across the two compared individuals). Specifically, we looked at three comparisons within each group, that is, three MZ twin comparisons, three parent–child comparisons and three unrelated pair comparisons. This calculation was used to test the relationship within each group in relation to their expected genetic relatedness. To perform the reciprocal overlap formula, HD-CNV was used with 50 as the identified RO merge criteria [26]. The *d* value was averaged for each type of relatedness in each individual software program. The results were assessed to compare the effectiveness of each individual algorithm in the identification of biologically relevant CNVs.

## Results and discussion

Supplementary Table S1 (see Additional File 1) shows the total number of unmerged CNVs representing gains and losses identified by each of the four software programs for 16 individuals using the same CEL files from Affymetrix^®^ Human SNP 6.0 Arrays™. The 16 individuals included in this analysis represent six pairs of MZ twins and the two parents for twin pairs 2 and 3. The results show that the number of raw CNVs identified in each individual varies depending on the program used. This variability is apparent in the numbers of gains and losses as well as the total numbers. Although AGC, PGS, and PC identified similar numbers of CNVs for most individuals (average of ~78 CNVs per individual), GH yielded more CNVs in each individual (average of ~317 CNVs per individual). While PGS yielded relatively more gains than losses, the other programs (AGC, PC and GH) yielded relatively more losses than gains. The differences in the number of gains and losses called between programs have suggested that each method may highlight some aspects of CNV calling but not others. We attempted to gain an insight into this variability by assessing the distribution of CNV calls in different contexts.

**Figure 1:**
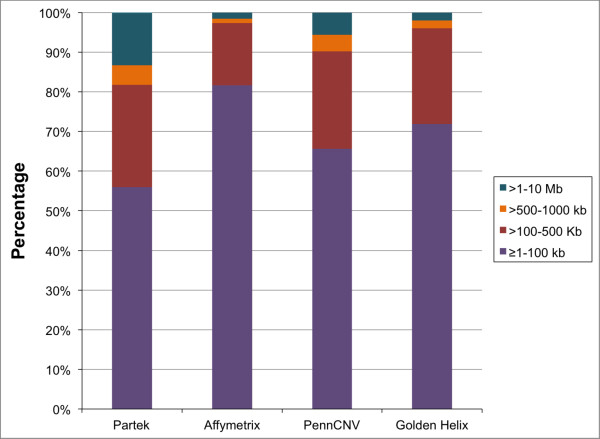
**Raw copy number variant calls by size. **Size distribution of 3957 raw copy number variants across six pairs of monozygotic twins.

**Figure 2:**
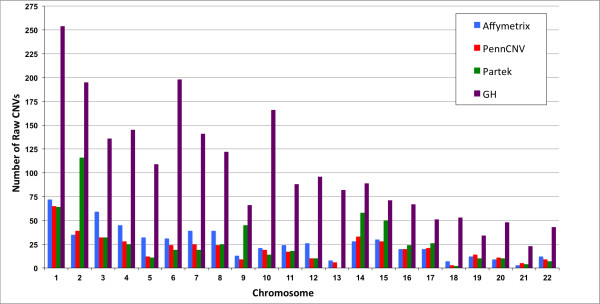
**Raw copy number variant chromosomal distribution. **Distribution of 3957 raw copy number variants in 22 autosomes across six pairs of monozygotic twins.

First, we assessed the size distribution of CNVs called by the four programs. We found that the four programs vary in the number of CNVs called and that CNVs fall into different size categories (Figure 1). CNVs in the range of 1-100 kb were most frequent in AGC calls (>80% of total calls) and least frequent in PGS calls (<60% of total calls). Similarly, the largest CNVs (1-10 Mb) were observed at higher frequency in PGS calls (>10%) as compared to the other three programs (range 1-5%). The chromosomal distribution of CNVs identified by the four programs showed that GH calls more CNVs on all chromosomes as would be expected with the higher number of calls overall (Figure 2). Also, this number is closely followed by PGS calls particularly on chromosome 2, 9, 14 and 15. Otherwise, the distribution of CNVs across chromosomes is proportional to chromosome size, as expected.

**Figure 3:**
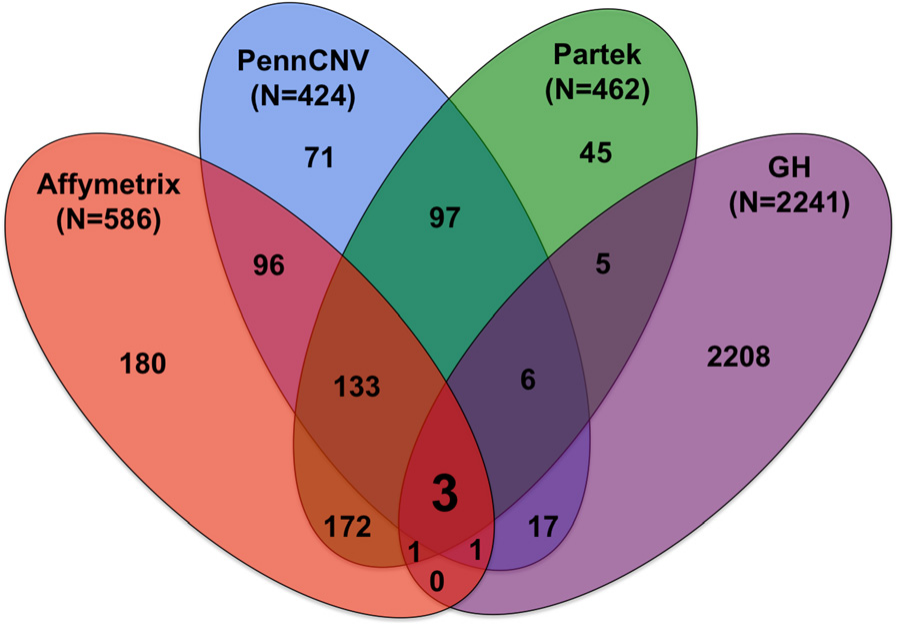
**Overlap of copy number calls across programs. **Venn diagram showing CNV (post merging of adjacent calls into one event) calls made by each software program across six pairs of monozygotic twins (numbers in parenthesis), as well as the number of CNVs shared in common between three software programs and between four software programs (3 CNVs). The total number of non-unique CNVs identified post-merge across four software programs was 3713.

Next, we assessed the overlaps in CNV calls made by each of the four programs in the twelve monozygotic twins (Figure 3). Most CNVs called by GH were not shared by calls made by any other program. The low degree of overlap suggests that the underlying assumptions of CNV calling by GH are different from the other three methods. Also, a significant number of CNV calls called by the other three methods (AGC, PGS and PC) showed overlaps. The CNV calls by PGS and AGC showed the most overlap (59%), closely followed by the overlap between PC and PGS (54%), and between AGC and PC (46%). CNV calls that overlapped between AGC, PGS, and PC represented 27% of the total number of CNV calls made by the three programs. When calls made by GH were included, all four programs shared only 0.32% (12/3713) of total CNV calls made. These results are similar to other reports involving comparison of different CNV calling programs [15,16].

**Figure 4:**
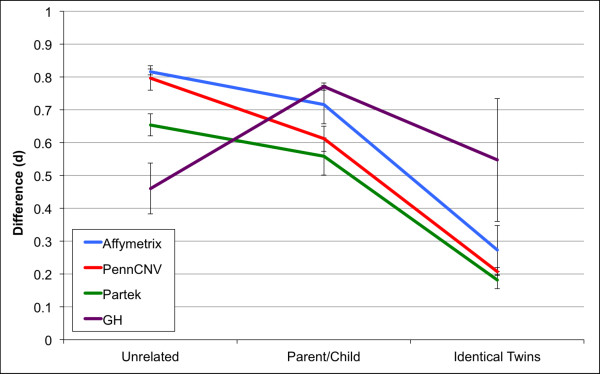
**Copy number call differences categorized by relatedness. **Mean difference (d) ± SEM between three pairwise comparisons between two unrelated individuals, parent and child, and monozygotic twins as determined by each software program (PennCNV, Affymetrix: Affymetrix Genotyping Console, Partek: Partek Genomics Suite, GH: Golden Helix SNP and Variation Suite).

We expected that differences and similarities in CNV calls would follow the genetic relatedness of individuals. For example, monozygotic twins would be expected to share the most CNV calls, while CNV calls for two unrelated individuals will show the highest amount of divergence. Similarly, pairs of individuals (parent and child) with presumed genetic relatedness would be expected to fall between 100% (MZ twins) and 0% (unrelated individuals). Figure 4 shows the degree of CNV difference *d* involving randomly selected pairs of unrelated individuals (n = 3), parent–child pairs (n = 3), and MZ twins (n = 3) for each of the four software programs. The estimate of difference (*d*) for CNVs called by GH has no relationship to the genetic relatedness between individuals. Conversely, CNV calls made by the other three programs (AGC, PGS, and PC) follow the known genetic relatedness. The largest difference between unrelated individuals was identified by AGC (82%) and followed closely by PC (80%). The smallest difference found between MZ twins is reflected by PGS (18%) followed by PC (21%). Interestingly, the parent–child differences for the three methods PGS, PC, and AGC were estimated to be ~56%, ~61%, and ~72%, respectively. Even though the standard deviations associated with these means (based on only 3 comparisons) are relatively large, the overall trends for the pairwise comparisons made by PGS, PC, and AGC follow the expected pattern based on the known genetic relatedness. These findings, in combination with the relatively high degree of overlap in CNV calls among these methods, support the likelihood that PGS, PC, and AGC are identifying biologically relevant CNV calls. Calls made by all programs provide a greater likelihood that the underlying CNV may be real. At the same time, if one is forced to choose only one method, our analysis based on the ability to resolve varying degrees of genetic relatedness favours the use of PC. The reason for this choice is based on the fact that it has a relatively high *d* value for unrelated individuals, a low *d* value between MZ twins, and an intermediate degree of difference involving parent and child, as is expected.

We compared four programs to call CNVs (AGC, PGS, PC, and GH) from the same microarray data for 16 individuals using the Affymetrix^®^ Human SNP 6.0 Array™. The CNV calls are different across the four methods, but some overlap was observed. It follows a number of other reports in the literature that have also reported similar discrepancies [27]. It is therefore not surprising that the results are different across the four methods and particularly between the three methods that use a HMM (AGC, PGS, and PC) versus the method that uses a segmentation approach (GH). Unlike HMMs that assume the means of different copy number states to be consistent, optimal segmenting delineates CNV boundaries with increased sensitivity. Overall, we have found that some CNV calling methods can appropriately distinguish known levels of genetic relatedness and some have more difficulty doing so. We also note that some differences between related individuals, including monozygotic twins would be expected due to somatic mosaicism; however, these differences would be expected to be relatively small in number.

The results presented in this report suggest that microarray experiments are prone to errors in CNV calls. Further, our results are likely to include false positive as well as false negative calls found in our arrays. Also, many of the CNV databases populated with entries over the past half-decade may include results that have not been confirmed [7,27-29]. In fact, research involving CNV calling from microarray results would benefit from better microarray technologies, better algorithms for CNV calling and better methods for independent confirmation.

All steps in a microarray experiment, from the isolation of DNA from tissue samples to the calling of CNVs from CEL files, are points at which error can be introduced. These and other confounding factors could affect the accuracy of biologically-significant CNV detection. Inferring copy number from array data is notoriously plagued with high false-positive rates that may vary depending on the algorithm used [27]. Given the clinical implications for accurate CNV detection [19] as well as the introduction of ascertainment bias into future studies via microarray design, algorithm parameters, and database entries [30,31], it is necessary to identify well-performing CNV calling programs.

## Conclusions

The results presented here offer a number of insights with respect to the total number of CNVs across individuals including gains and losses, program-specific distributions of CNV size, and CNV distribution across chromosomes. As expected, the number of CNVs was related to the size of the chromosome. The major observation from the collective results is that the number of CNVs identified by Golden Helix (GH) software was much higher than the number called by the other three programs (AGC, PC, PGC). Also, Golden Helix identified vastly different CNVs when compared to those identified by Affymetrix^®^ Genotyping Console™ (AGC), Partek^®^ Genomic Suite (PGS), and PennCNV (PC). Excluding GH as an outlier, AGC yielded more CNV calls than PGS and PC. As expected, AGC, PGS, and PC yielded a relatively large number of overlapping CNVs. These results are consistent with a number of reports including Pang et al. [23].

Further assessment of the four CNV calling methods was considered in the context of similarities and differences involving individuals of differing genetic relatedness. As it stands, three of the four methods met such expectations to different degrees. The results offer two conclusions. First, overlapping CNV calls by three of the programs (AGC, PGS, and PC) will offer the highest likelihood of discovering biologically relevant calls as compared to any other group of the four programs used in this report. The combination of AGC and PC identified the most differences among unrelated individuals whereas PGS and PC showed the least differences between MZ twins. The results from GH showed a higher number of CNVs than would be expected and also did not follow the expected pattern when groups of known relatedness were compared. For this reason, we do not recommend GH and suggest that further research should explore the unexpected profile generated from the software. Secondly, the PC calls best reflect the expectations at all three levels involving unrelated individuals, parent–child, and MZ twins. Our results and conclusions support other groups, which have found that without independent validation using bench confirmation techniques such as qPCR, CNVs calls should not be assumed to be truly valid variants [16,32]. Finally, we suggest that incorporation of family data will help in improving the quality of CNV calls alongside the use of multiple CNV calling methods.

### Availability of supporting data

The data set supporting the results of this article is available in the Gene Expression Omnibus (GEO) repository, [GSE33598, http://www.ncbi.nlm.nih.gov/geo/query/acc.cgi?acc=GSE33598] [33].

## Abbreviations

AGC: Affymetrix^®^ Genotyping Console 4.1.1; CNV: Copy number variant; DGV: Database of Genomic Variants; GH: Golden Helix^®^ SVS Suite 7.0™; HMM: Hidden Markov Model; PC: PennCNV; PGS: Partek^®^ Genomics Suite™; RO: Reciprocal overlap.

## Competing interests

The authors declare that they have no competing interests.

## Authors’ contributions

SS, RO, and CC conceived and designed the experiments. CC, MM, AW, MEO, and ZA performed the experiments. CC and AW analyzed the data. CC and SS wrote the paper. All authors read and approved the final manuscript.

## Supplementary Material

Additional file 1: Table S1 Raw Copy Number Calls by Program. Number of raw (pre-merge) copy number variant calls in six pairs of monozygotic twins and two sets of parents. Click here for file
