# Supplementary material for: Biological relevance of CNV calling methods using familial relatedness including monozygotic twins
Source: BMC Bioinformatics. 2014 Apr 21;15:114. doi: 10.1186/1471-2105-15-114 (PMC4021055; doi:10.1186/1471-2105-15-114)
Supplement: Additional file 1: Table S1 — Raw Copy Number Calls by Program. Number of raw (pre-merge) copy number variant calls in six pairs of monozygotic twins and two sets of parents. [file 1471-2105-15-114-S1.pdf]

Supplementary Table 1. Raw Copy Number Calls by Program. Number of raw (pre-merge) copy number variant calls in six pairs of monozygotic twins and two sets of parents.

ID	Affymetrix Genotyping Console			PennCNV			Partek Genomics Suite			Golden Helix SVS		
	Gain	Loss	Total	Gain	Loss	Total	Gain	Loss	Total	Gain	Loss	Total
**1A**	12	29	41	12	24	36	22	31	53	47	121	168
**1B**	19	31	50	20	21	41	44	28	72	107	102	209
**2A**	19	29	48	17	24	41	49	17	66	79	121	200
**2B**	17	29	46	17	25	42	41	24	65	77	114	191
**3A**	19	28	47	9	16	25	34	20	54	72	120	192
**3B**	18	26	44	6	15	21	29	14	43	73	127	200
**4A**	13	31	44	7	18	25	36	13	49	78	122	200
**4B**	17	35	52	6	16	22	25	12	37	63	107	170
**5A**	15	41	56	8	37	45	15	39	54	82	122	204
**5B**	12	42	54	7	34	41	13	45	58	73	99	172
**6A**	30	31	61	25	25	50	20	27	47	82	104	186
**6B**	26	27	53	26	27	53	21	27	48	73	108	181
**Father 2A/2B**	1065	101	1166	49	42	91	105	32	137	1009	297	1306
**Mother 2A/2B**	175	34	209	13	35	48	19	96	115	496	301	797
**Father 3A/3B**	24	41	65	14	13	27	16	11	27	113	263	376
**Mother 3A/3B**	21	48	69	10	25	35	9	89	98	62	262	324
